# Prevalence of lung atelectasis in sedated dogs examined with computed tomography

**DOI:** 10.1186/s13028-022-00643-0

**Published:** 2022-09-08

**Authors:** Elin Reimegård, Helena T. Nyman Lee, Frida Westgren

**Affiliations:** Department of Diagnostic Imaging, Evidensia Södra Djursjukhuset, Månskärsvägen 13, Kungens Kurva, 141 75 Stockholm, Sweden

**Keywords:** CT-scan, Intravenous sedation, Lung attenuation, Sternal recumbency

## Abstract

**Background:**

Computed tomography (CT) scanning of the lung is known to be a valuable tool when investigating lung pathology of the dog. During CT-scan the dog needs to be immobilized and general anesthesia has historically been considered as gold standard although being a more expensive and time-consuming alternative to sedation. Today, modern high speed multidetector CT-scanners offer new possibilities for sedation as an alternative. Both anesthesia and sedation can cause lung atelectasis, and this can be problematic when reading the CT-images since it potentially can masque or mimic lung pathology leading to misdiagnosis. The objective of this prospective analytic study was to investigate the prevalence of lung atelectasis and changes in lung attenuation over time in dogs that receive intravenous sedation and positioned in sternal recumbency.

**Results:**

20 dogs without known lung pathology underwent three consecutive CT-scans of the lung; the first scan was initiated as soon as the dog was sufficiently sedated, the second scan approximately 5 min after the first one and the last scan after the dog’s orthopaedic scan was completed. The dogs received intravenous sedation in a combination of dexmedetomidine and butorphanol and were kept positioned in a strict sternal recumbency during sedation and exam. Each lung lobe was individually examined in an axial plane and measurements of dorsal, ventral, and mean lung attenuation were made. Atelectasis or areas with poorly aerated lung tissue were not detected as all parts of the lobes were normally aerated at all three scans. A statistically significant increase in lung attenuation between the first and the second scan (P = 0.03) and between the first and the third scan (P = 0.0004) was seen in the ventral part of the lobes.

**Conclusions:**

This study indicates that CT-examination of the lungs can be performed on sedated dogs that are kept in a sternal recumbency without development of atelectasis. It also suggests that there is an early correlation between time and increase in lung attenuation.

## Background

Computed tomography (CT) scanning is known as a valuable tool when examining thoracic and pulmonary disease in dogs and cats and is considered gold standard for diagnosing several conditions [1–4]. Animals undergoing CT-scans generally need to be sedated or anesthetized in order to perform the exam, but this may increase the risk of developing an increase in lung attenuation and even lung atelectasis [5–9]. Lung atelectasis is defined as an value between − 100 to 100 Hounsfield units (HU) while increase in lung attenuation ranges between − 500 to − 101 HU [15].

Lung atelectasis can be problematic when interpreting the images for mainly two reasons: firstly, atelectasis may mask true pathology. Secondly, there is a risk of misinterpreting atelectasis as a pathological change in the lung parenchyma.

Although successful CT studies of awake animals have been done using positioning devices in cats where little or no development of lung atelectasis was seen, [3, 10], this is not applicable in every day practice, especially not in dogs with high stress levels. Previous CT´studies have evaluated how lung attenuation alters with different anesthesia protocols and recumbencies [6–8, 11–13]. Results point towards the fact that animals positioned in a lateral recumbency tend to be at a higher risk of developing atelectasis or increase in lung attenuation than animals positioned in a sternal recumbency [7, 13, 14]. These changes tend to develop rapidly after anesthesia is induced, and that once it occurs, it might not resolve although the animal is repositioned [7]. In one recent study [9], ten dogs receiving intravenous sedation were examined with CT scanning showing no atelectatic lung changes. This study also showed that approximately 13,1% of the dogs developed hypoinflated lung parenchyma of which 2,4 ± 1,2% had a lung attenuation greater than − 250 HU.

The aim of this study was to determine if dogs without known lung pathology that were sedated intravenously with a combination of dexmedetomidine and butorphanol and positioned in sternal recumbency develop lung atelectasis. Alteration of lung attenuation over time was also assessed.

## Methods

### Dog population

This study took place at Evidensia Animal Hospital Södra Djursjukhuset (“South Animal Hospital”), Stockholm, during the period August 2019 to August 2020. Dogs included in this study were selected from a larger group of dogs undergoing a CT examination for front limb orthopaedic disease. The specific group was selected because they receive sedation as a standard, and they are examined in a sternal position, which was two important criteria for this study. None of the dogs received any intravenous contrast medium as a part of their orthopaedic limb CT-exam.

Dogs with lung disease, signs of systemic infection or inflammation according to blood test results and clinical examination, brachycephalic breeds, and dogs with recently known trauma or with suspicion of neoplasia were excluded. All dogs underwent physical examination prior to the CT-scanning including cardiac and pulmonary auscultation, heart rate, respiratory rate, assessment of general condition, and examination of mucus membranes. Basic parameters such as age, breed and gender were obtained from the patients’ journal. Blood samples were collected at the day of the study and C-reactive protein (CRP) and complete blood count was analyzed. Animals with hematologic signs of systemic infection or inflammation were excluded.

### Study design

All animals received an aseptically peripheral vein catheter placed into a cephalic vein before receiving any sedatives. Sedation was given intravenously using the following protocol: butorphanol (Butomidor vet^®^ 10 mg/mL) 0.2–0.4 mg/kg in combination with dexmedetomidine (Dexdomitor^®^ 5 mg/mL) 2.5–5 mcg/kg followed by sterile saline to flush the syringe. If additional sedation was needed, this was also administered intravenously. Sedatives were given directly prior to the first CT scan. The dog was positioned in sternal recumbency with front legs pulled forward, and head in between its front legs.

**Fig. 1:**
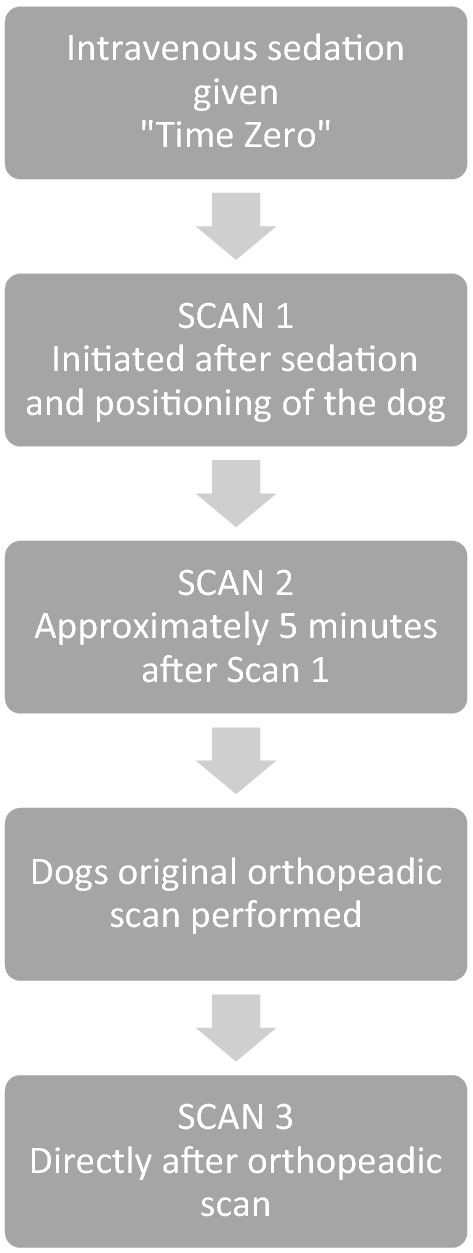
Flow chart showing the onset of the three different lung scans; Scan 1, Scan 2 and Scan 3. Time zero stated as the time of sedation

Three lung scans (Scan 1—3) were performed on each patient. Time zero was stated as the time of sedation. According to Fig. 1, Scan 1 was performed as soon as possible after the patient was sufficiently sedated and correctly positioned, Scan 2 as shown approximately 5 min later. The ordinary scan, meaning the designated orthopaedic scan of the patient, was then performed prior to the third delayed scan, Scan 3, which could vary in time depending on how long time the ordinary scan took. A protocol was used to note the time for all three scans (Table 2).

### CT examination and interpretation

All dogs were scanned in the same scanner (Philips Ingenuity Core 128 powered by iPatient, scanner V4.1.7.10503, Philips Healthcare Netherland B.V, Veenpluis 4–6, 5684 PC Best, Netherlands) 128 slice. A standardized lung protocol was used with technical parameters as follows: slice thickness 1.0 mm, pitch 1.074, matrix 512, image field of view − 600 to 1600, lung reconstruction algorithm Y-Detail (YD), kV 100, mAs 182, rotation time 0,75. Window width and level could be adjusted between study animals. Scans were obtained in a cranial to caudal direction with the dog in a sternal position. The time required for doing a lung scan using the dedicated machine varies between 3 to 10 s depending on the dog’s size.

Images were viewed in a PACS working station using the same software program for measurements of all dogs (HOROS™ dicom viewer). All images were analyzed by a single observer (aspirant in the Swedish program for Specialist in Diagnostic Imaging) under supervision of a board-certified specialist (Dipl.ECVDI).

Each lung lobe: left cranial lobe pars cranialis (LCPCr) and pars caudalis (LCPCa), left caudal (LC) lobe, right cranial lobe (RCr), right middle lobe (RM), right caudal lobe (RCa) and the accessory lobe (LA) were individually examined at all three scans. An axial slice where the most ventral tip of the lobe was included, was chosen and images from the three scans were compared and propagated so that measurements were done at the same topographic level on all time intervals. If the dog was repositioned between the lung scans, anatomical landmarks such as vertebrae, bronchi and vessels were used to examine the lobe at the same level. Care was taken that the most ventral tip of the lung lobe was included in the slice since lung atelectasis was expected to develop at this level first [15].

**Fig. 2:**
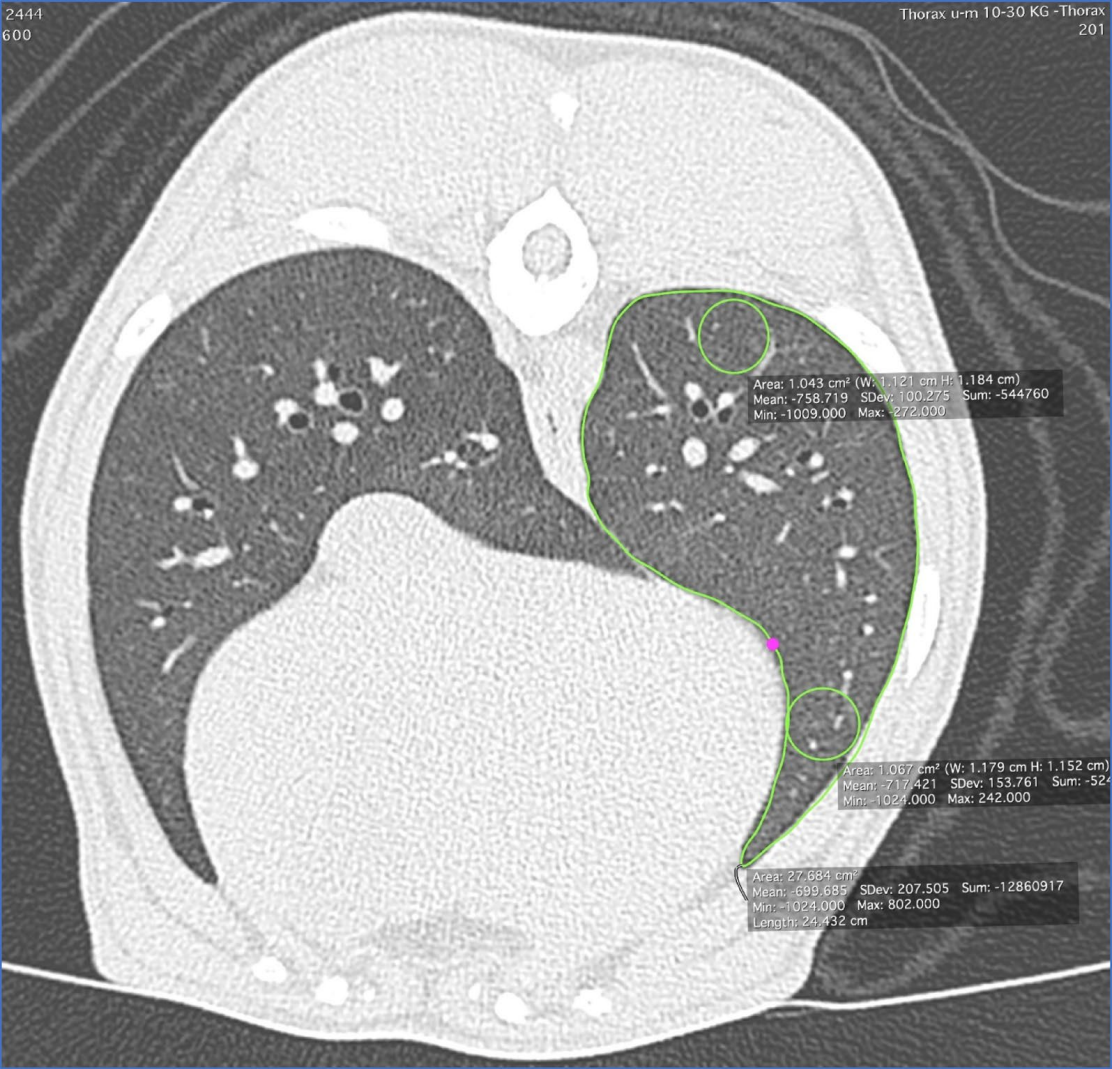
Axial slice showing how measurements were done in each lobe, in this image represented by the left caudal lung lobe. Lung attenuation was measured by placing a ROI of a minimum of 1 cm in the most dorsal and ventral part of the lung lobe, a mean was measured by drawing out the margins of the lung lobe using a free hand drawing tool

Three measurements of lung attenuation measured in HU were collected for each lobe (Fig. 2). First, a mean attenuation for each lung lobe was determined by manually drawing out the outlines of the lung lobe using a software tool included in HOROS™, excluding the main bronchi and aligned vessels. Second, a region of interest (ROI) of a minimum of 1 cm in diameter was manually drawn in the most dorsal part of the lung lobe. And third, a circle of ROI of a minimum of 1 cm in diameter, was drawn at the most ventral part of the lobe. Care was taken to avoid larger blood vessels and bronchi and the same anatomical areas were included in all of the three scans. If areas of visually increased attenuation could be seen these were also included using a free hand circle of ROI.

Lung attenuation was measured in HU and following values were used for interpreting the degree of lung attenuation: nonaerated (− 100 to + 100 HU) indicative of atelectasis, poorly aerated (− 500 to − 101 HU), normally aerated (−  900 to − 501 HU) or hyperaerated (− 1000 to − 901 HU) [12].

### Statistical analyses

The statistical analyses were carried out in Matlab R 2020a.

To determine if there was a statistically significant increase in lung attenuation over time, a paired test “Signed rank Wilcoxon test” was used. For each individual, a mean of the ventral attenuation values of Scan 1 and scan 2 respectively Scan 1 and Scan 3 were compared.

## Results

### Animals

During the chosen period, 20 dogs that fit the inclusion criteria were examined (Table 1). All dogs recovered uneventfully from sedation.

**Table 1:** Basic data of study group including sex, breed and age

Dog	Sex	Breed	Age
1	M	Bernese mountain dog	8 months
2	M	Labrador retriever	4 years
3	M	Labrador retriever	10 months
4	F	Australian shepherd	1 year
5	FN	Hovawart	11 months
6	M	Miniature poodle	8 years
7	M	Golden retriever	1 year
8	M	Nova scotia duck tolling retriever	5 years
9	F	Labrador retriever	1 year
10	F	Rottweiler	10 months
11	M	White shepherd dog	1 year
12	M	Staffordshire bullterrier	8 years
13	M	German shepherd	1 year
14	FN	Mixed breed	4 years
15	F	Shappendoes	1 year
16	M	Labrador retriever	1 year
17	M	Dogue de bordeaux	3 years
18	M	German shepherd	1 year
19	F	Rottweiler	8 months
20	F	Staffordshire bullterrier	1 year

*M* male, *F* female, *FN* female neutered

### Clinical exam and blood sample

All dogs were clinically unapparent and without findings indicating systemic infection or inflammation, made by hematology and biochemistry.

Two dogs (Nos. 2 and 9) had a slight elevation in CRP 23.6 and 28.7 mg/L, respectively (reference interval 0–20 mg/L). In these dogs, there were no elevation of white blood count or signs of toxic neutrophils or left shift, and the dogs had normal clinical parameters.

### Scan 1, 2 and 3

The different times for initiation of Scan 1,2 and 3 for each individual is presented in Table 2 with time zero stated as the time of sedation Fig. 2.

**Table 2:** The time noted for the three separate lung scans (Scan 1, Scan 2, Scan 3) for all 20 dogs, with time zero stated as the time of sedation

Dog	Scan 1	Scan 2	Scan 3
*1*	3 min 34 s	5 min 11 s	10 min 53 s
*2*	3 min 41 s	8 min 58 s	16 min 22 s
*3*	7 min 50 s	12 min 50 s	18 min 50 s
*4*	6 min	11 min	13 min 52 s
*5*	4 min 37 s	9 min 50 s	15 min 10 s
*6*	3 min 30 s	9 min	16 min 40 s
*7*	3 min 59 s	5 min	10 min
*8*	3 min 40 s	8 min 40 s	16 min 24 s
*9*	4 min 42 s	9 min 50 s	20 min 4 s
*10*	3 min 44 s	8 min 44 s	14 min 39 s
*11*	4 min 12 s	9 min 12 s	22 min 48 s
*12*	5 min 20 s	10 min 20 s	21 min 15 s
*13*	4 min 15 s	9 min 15 s	19 min 15 s
*14*	3 min 40 s	8 min 40 s	24 min 10 s
*15*	3 min 30 s	5 min 30 s	13 min 26 s
*16*	7 min 13 s	12 min 13 s	20 min 8 s
*17*	4 min 43 s	9 min 43 s	17 min
*18*	6 min 52 s	11 min 52 s	20 min 20 s
*19*	4 min 19 s	5 min	9 min
*20*	5 min 10 s	10 min 10 s	19 min 40 s

### Technical data

**Fig. 3:**
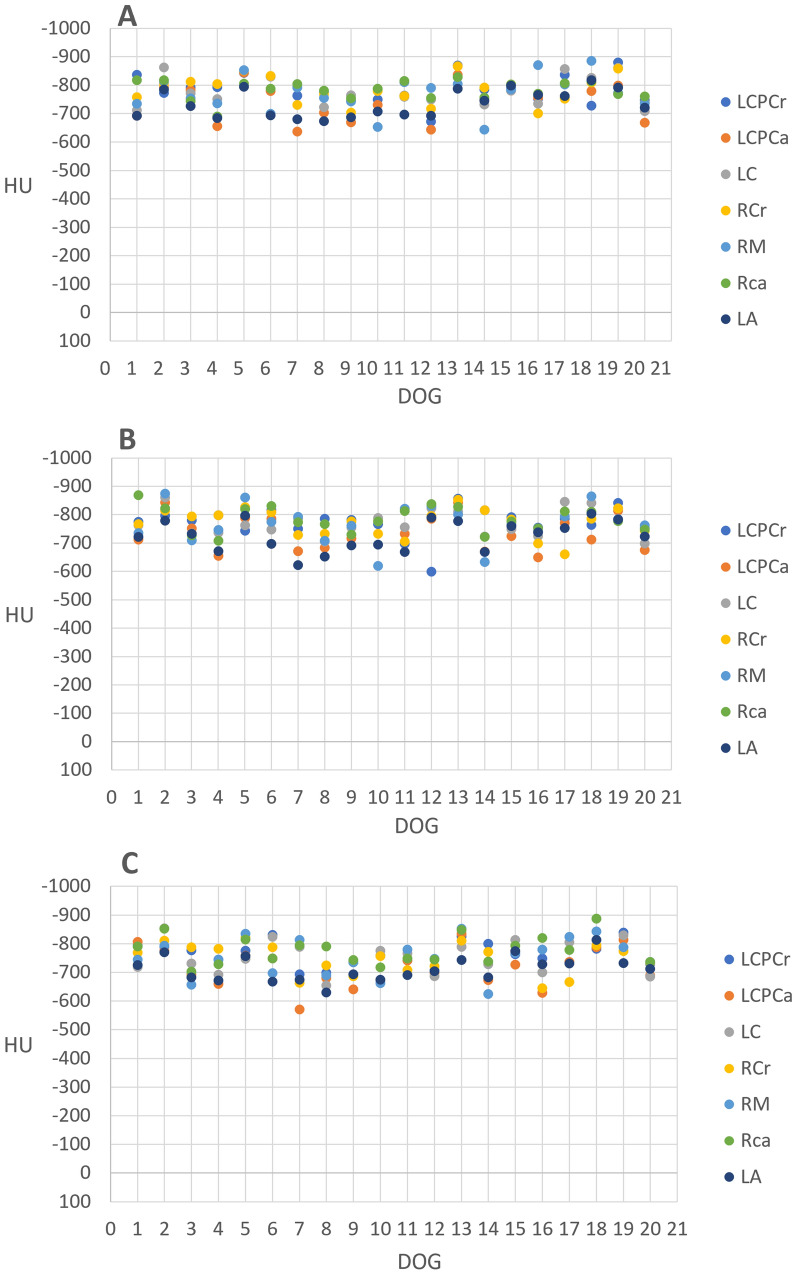
Lung attenuation in the ventral part of each lung lobe at Scan 1 (**A**), Scan 2 (**B**) and Scan 3 (**C**) measured in HU (y-scale). Values were obtained by drawing a circle of ROI in the ventral part of each lung lobe. Each colored dot represents an individual lung lobe for study dog 1–20 (x-scale)

The results showed that all measured areas of the lung parenchyma, both mean and the dorsal and ventral ROI-measurements were within the range of normally aerated lung parenchyma, defined as − 501 to − 900 HU (Fig. 3A–C). Hence, no atelectasis (− 100 to + 100 HU) or areas with poorly aerated lung tissue (− 500 to − 101 HU) were detected.

Though all measurements were within the range of normally aerated lung tissue, a diversity within this spectrum could be seen. The highest mean quantitative measurement was found in the left cranial lobe pars caudalis (LCrPCa) with a value of − 569 obtained at the third scan (Scan 3) of dog nr 14. This third scan was also initiated last of all scans (24 min and 10 s after sedation). The highest quantitative focal measurement done with ROI was from the ventral part of the LCrPCa with a value of—571 HU obtained at the third scan (Scan 3) of dog no. 7. A motion artefact was noted from the same scan (Scan 3, dog no. 7) and this was the only motion artefact noticed.

**Fig. 4:**
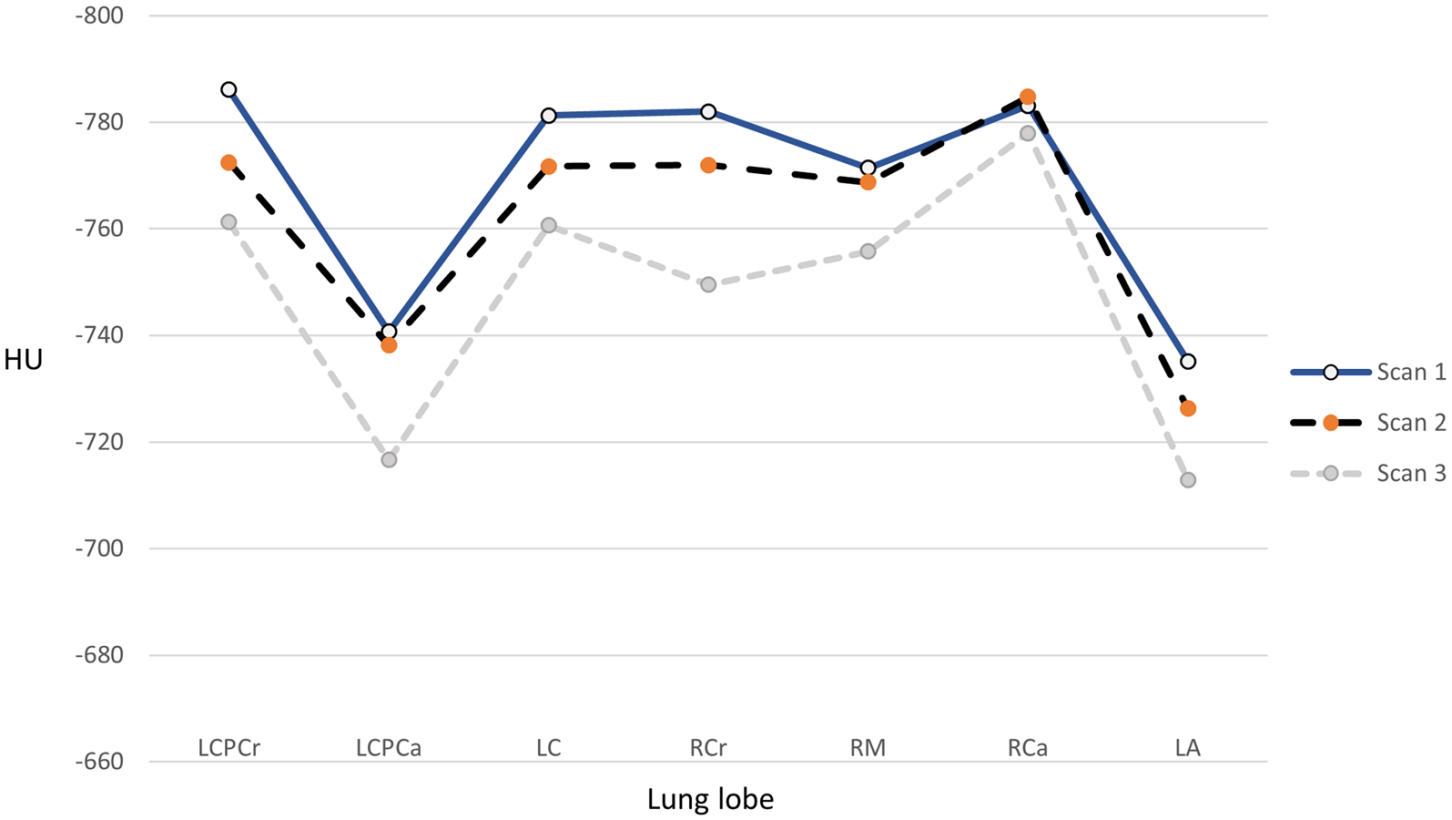
Mean value of ventral part of each lung lobe measured at Scan 1, Scan 2 and Scan 3

Comparison of measurements between the different lung lobes at Scan 1, Scan 2 and Scan 3 is illustrated in Fig. 4, indicating an overall increase in lung attenuation over time. This association was further investigated with statistical analyses described below.

**Fig. 5:**
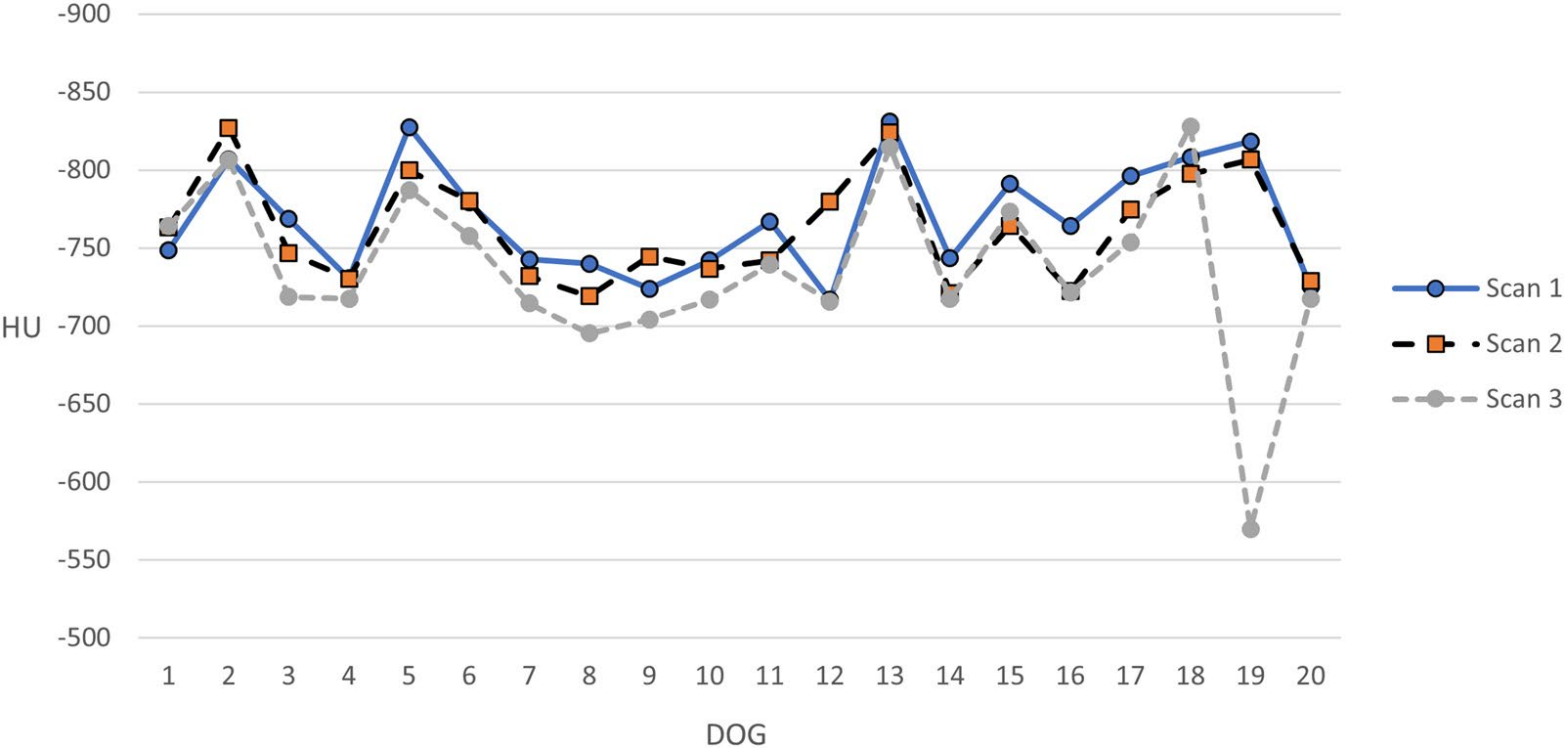
Mean lung attenuation as measured from the ventral part of all lobes for individual 1–20 at Scan 1, Scan 2 and Scan 3

For each individual we computed the total lung attenuation as mean Hounsfield value across all lobes, as measured in the ventral part of the lobe (Fig. 5). A one-sided Wilcoxon signed rank test showed that the median total lung attenuation was significantly greater at scan 2 than at Scan 1 (P = 0.03), and greater at Scan 3 than at Scan 1 (P = 0.0004).

## Discussion

The aim of this study was to investigate if sedation was associated with lung atelectasis in dogs undergoing CT scanning. The findings show that neither atelectasis nor areas with poorly aerated lung tissue could be detected. These findings strengthens the results from a recent study done on sedated animals examined with CT [9], indicating that sedation alone is not a risk factor for lung atelectasis in healthy dogs. Also, compared to previous studies done on anesthetized animals, these results show lower frequency of alterations in lung attenuation [3, 8, 11, 14].

Although all measurements were within the range of normally aerated lung tissue, a statistically significant correlation between time and an increase in lung attenuation in the ventral part of the lung was observed. These changes developed rapidly between Scan 1 and Scan 2. These findings support previous studies in which alterations in lung attenuation developed rapidly, often within 3 to 8 min after anesthesia, and then remained relatively static over time [7–9]. The findings indicate that it is beneficial to initiate the patient’s CT scanning as soon as possible after sedation or anesthesia is induced to avoid an increased lung attenuation. In this study, all 20 dogs initiated their first scan within 7 min and 50 s after sedation and all scans were completed within a time span of up to 24 min and 10 s. This shows that if the patient and the staff are well prepared, it is possible to obtain a fast CT scanning of the lungs and still have time to perform additional scans such as contrast studies before any clinically significant increase in lung attenuation develop.

The highest mean quantitative attenuation measurement of a lobe was found in LCrPCa with a value of − 569 obtained at the third scan (Scan 3) from dog no. 14. This scan was initiated 24 min and 10 s after sedation, and this was also the one scan that was last initiated of all scans. The highest quantitative measurement obtained from a ROI, with a value of − 571 HU was made in dog no. 7 at the time of the 3rd scan. This was obtained from the ventral part of the left cranial lung lobe pars caudalis. The two measurements with the highest values were from the same lobe (LCrPCa) both obtained from the 3rd scan. These findings might indicate that there is an increased risk for increased lung attenuation in this lobe, potentially because of its small volume. Given the small number of animals used in this study, a statistically significant difference between lobes was not found.

Previous studies have shown that animals that are positioned in lateral recumbency are at risk of developing an increase in lung attenuation in the dependent lung lobe [7, 14, 15]. To avoid lateral or dorsal recumbency at any point and potentially resulting atelectasis, a fast intravenous administration method was chosen making it possible to support the animal in a sternal recumbency. For sedation, a combination of dexmedetomidine and butorphanol was used and injected intravenously with a rapid onset of sedation, making it possible to perform a first CT examination within a few minutes.

In anaesthetized animals the breathing can be controlled by using a breath holding technique [11] making it possible to avoid motion artifacts. When an animal is sedated using dexmedetomidine the respiratory rate is normally reduced [16] and this in combination with high scan speed in a modern multidetector CT scanner, provides the possibility to a large extent to avoid motion artefacts caused by breathing. Motion artefacts from breathing was only detected in one dog in one of the scans (Scan 3, dog no 7).

During the chosen period, 20 dogs that fit the inclusion criteria were included. Mainly young dogs are represented: 13/20 dogs were at the time of the study 1 year or younger and the oldest dog included was 8 years old. This is likely due to the high frequency of developmental orthopaedic disease affecting young individuals, as clinical indication for an orthopaedic CT exam was one of the inclusion criteria in the study.

This study was done on animals without clinical signs or any history of pulmonary disease where the described sedative protocol worked well. Due to this fact, it is possible that results from this study cannot be applicable on older animals or animals with lung disease where atelectasis might develop more easily.

A manual measuring method was chosen to determine the degree of lung attenuation by drawing the outlines of each lung lobe and also by using ventral and dorsal ROI. By using this method, all parts of the lung parenchyma independent of alterations in attenuation was included in the results. Since the HU-spectra for atelectasis, soft tissue and fat partly overlap, there could potentially be a risk of missing important areas of the lung parenchyma if a computerized method is used. When measuring the ventral and dorsal parts of the lung lobe, a ROI of a minimum of 1 cm in diameter was chosen. This was done in order to include an area large enough to minimize the alteration in attenuation caused by minor vessels and bronchi, but still be able to see focal differences between the dorsal and ventral parts of the lungs. In smaller dogs, this method was not optimal since the most ventral tip of the lobe was not included in the ventral measurements. Since this is the area where atelectasis could first arise, a mean attenuation including the tip of the lung was also obtained. Note should be taken that if a subjective increase in attenuation was seen, it would have been measured.

Measurements of lung attenuation was made in an axial plane in one representative slice for each lobe. It was not possible to standardize measurements to a certain lobe in relation to a certain vertebra because the individual variations between the study animals was too great leading to a subjective decision for where the most representative part of the lung was. When measuring a lobe at the three different time intervals, the images were linked to make sure that measurements were made in the same axial plane for the individual dog.

## Conclusions

Intravenous sedation in combination with sternal recumbency provides excellent quality in CT images with low risk of developing lung atelectasis or attenuated lung tissue in dogs without known lung pathology, up to 24 min after sedation. The results also indicate that an increase in lung attenuation develop within minutes after sedation and that rapid sedation and initiation of scanning is therefore advantageous.

## Data Availability

The datasets generated and analyzed during the current study are not publicly available due to regulations under the General Data Protection Regulations, but parts of the data may be available from the corresponding author upon reasonable request.
